# Supplementary material for: Assembly mechanism of the pleomorphic immature poxvirus scaffold
Source: Nat Commun. 2022 Mar 31;13:1704. doi: 10.1038/s41467-022-29305-5 (PMC8971458; doi:10.1038/s41467-022-29305-5)
Supplement: Supplementary file 1 — Supplementary Information [file 41467_2022_29305_MOESM1_ESM.pdf]

# Assembly mechanism of the pleomorphic immature poxvirus scaffold

J. Hyun, H. Matsunami, T.G. Kim and M. Wolf

## Supplementary Information

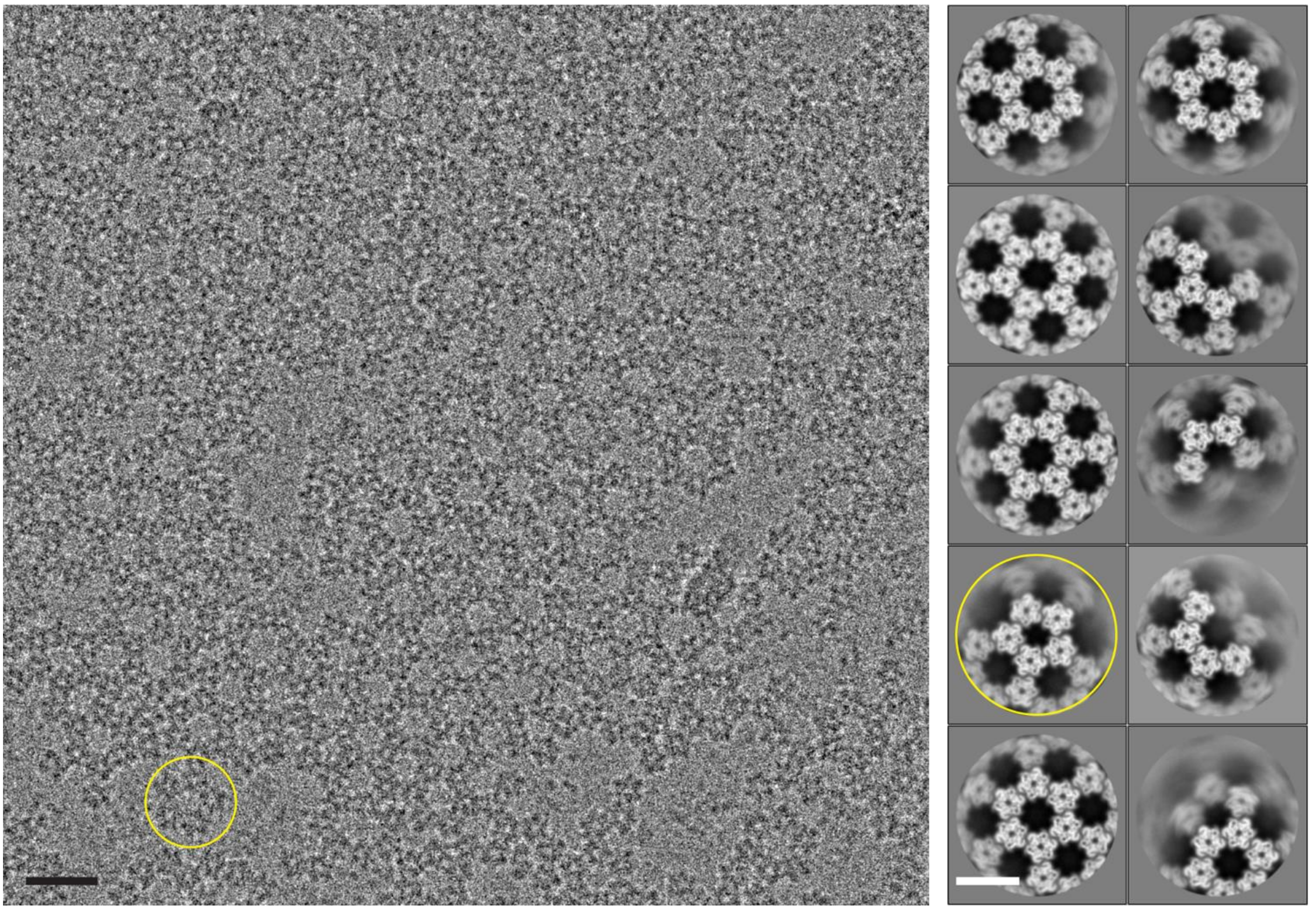

**Supplementary Figure 1. Variations in the building blocks of the honeycomb lattice**

Cryo-electron micrograph of purified D13 trimer vitrified on a holey carbon grid without additional graphene oxide support film (left) and representative class averages of rings of trimers (right). 52,935 particle images were used to generate the 2D class averages. Spontaneous *in situ* formation of honeycomb-like lattice patches was observed at the air-water interface when prepared without graphene oxide film (Supplementary Video 1). The majority of rings are composed of six trimers (trimer sextets) whereas rings of five trimers (trimer quintets) were also found (yellow circle). Scale bars, 20 nm and 10 nm in the micrograph and class averages, respectively.

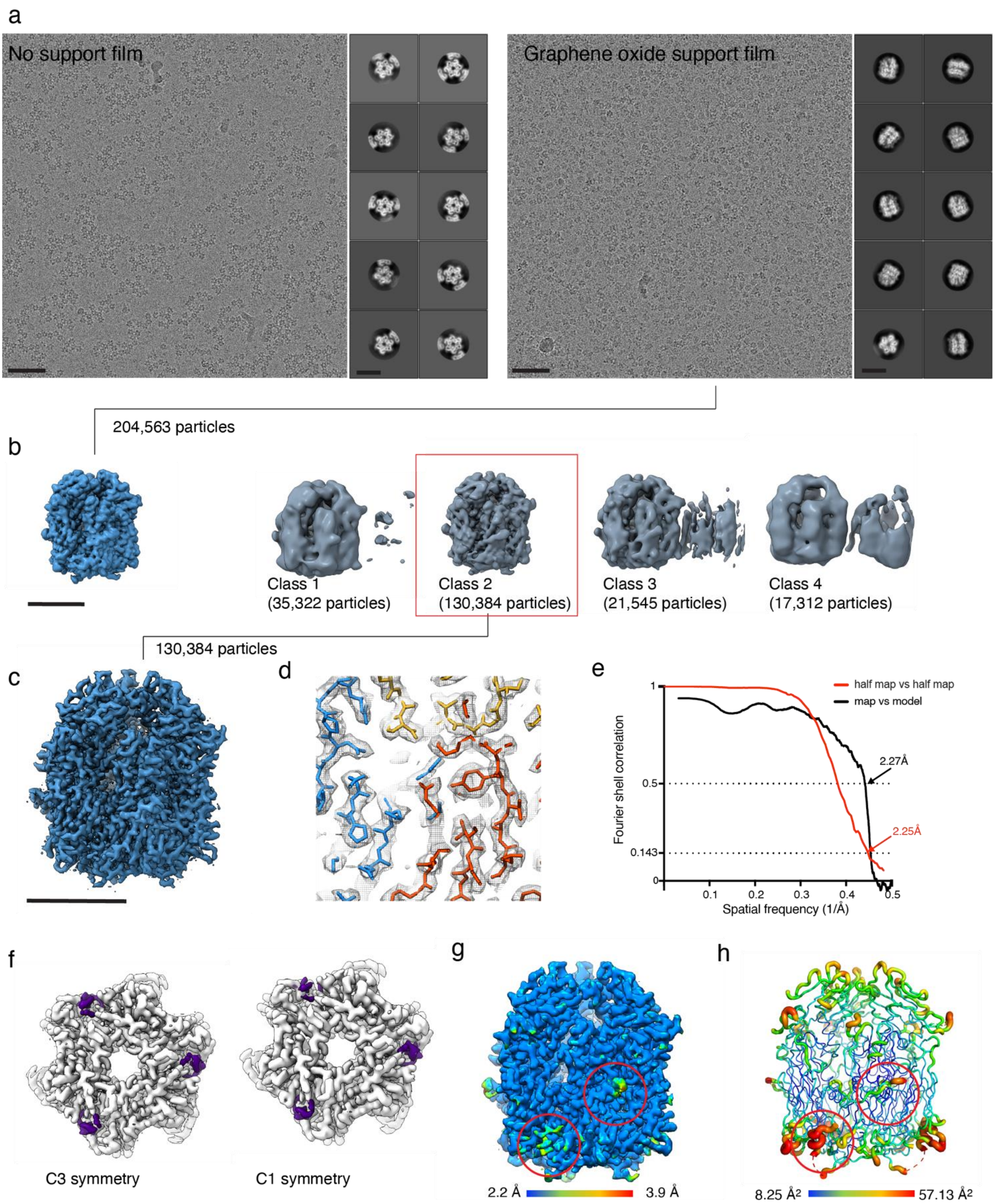


**Supplementary Figure 2. D13 trimer cryo-EM image processing**

**a**, A representative cryo-electron micrograph and 2D class averages (inset) generated from 47,289 and 204,563 images of the particles without (left) and with graphene oxide support film (right), respectively. **b**, 3D classification without symmetry imposition. Particles that belong to the best 3D class (Class 2) were selected for subsequent 3D refinement. **c**, Isoelectron potential surface contoured at 3.0σ of the final 3D reconstruction of the D13 trimer. **d**, Representative fitting of the atomic model into the cryo-EM map displayed as isoelectron potential a mesh surface contoured at 5.0σ above average. **e**, Fourier shell correlation between the half maps (red curve), and between

the final map and model-generated density (black curve). The FSC is truncated, because the reconstruction was binned. The curve reaches zero in the unbinned reconstruction. **f**, D13 trimer structure refined using C3 symmetry, or without symmetry imposition. The reconstruction is viewed from the base of the trimer along the symmetry axis. **g**, Local resolution estimation of C3 symmetry-imposed 3D reconstruction and **h**, variation of atomic B-factors assigned to each residue during model refinement (in tube representation), viewed perpendicular to the symmetry axis. Examination of the cryo-EM maps and the corresponding fitted atomic models indicated relatively poor local resolutions at the N-terminal loop and tail helix that participate in domain swapping between the monomers (aa 1-14), and at the loop that connects β-strands B and C of the N-terminal jelly roll domain (aa 46-49). This suggests increased flexibility of these structural elements (red circles). Unsharpened cryo-EM maps were used to maintain visibility of less resolved regions. The figure shows isoelectron potential surfaces contoured at 5.0σ above average potential. Scale bars, 50 nm in (a), 5 nm in the inset of (a), and 5 nm in (b, c), respectively.

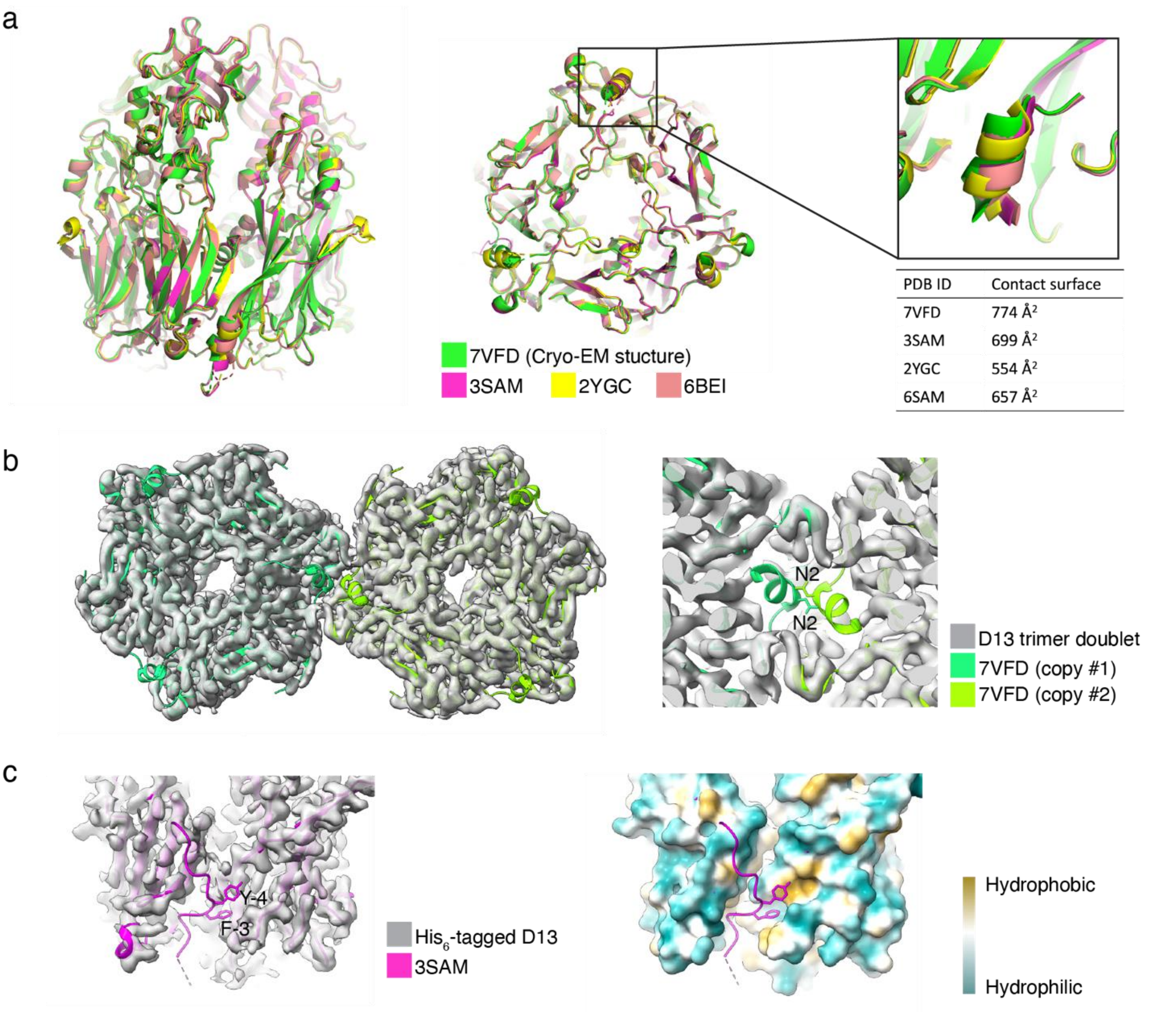


**Supplementary Figure 3. Structural examination of the N-terminal helix**

**a**, Superimposition of the atomic model of D13 determined by cryo-EM in this study (PDB 7VDF) with previous atomic models determined by X-ray crystallography, excluding structures in presence of rifampicin or rifampicin-derivatives. The positions of the N-terminal helices differ only slightly, with subtle changes in the contact surface area between the helix and their binding pockets. **b**, When the atomic models of D13 including N-terminal helix coordinates are docked into the cryo-EM map of the D13 trimer doublet, the helices clash at their overlapping N2 residues. **c**, Fitting of the X-ray crystal structure (3SAM) into the cryo-EM map of His$_6$-tagged D13, showing the N-terminal tag linker and its hydrophobic residues (Y-4, F-3) located in the hydrophobic pocket. The corresponding density is missing in the cryo-EM map, suggesting structural perturbation or displacement of the N-terminal helix.

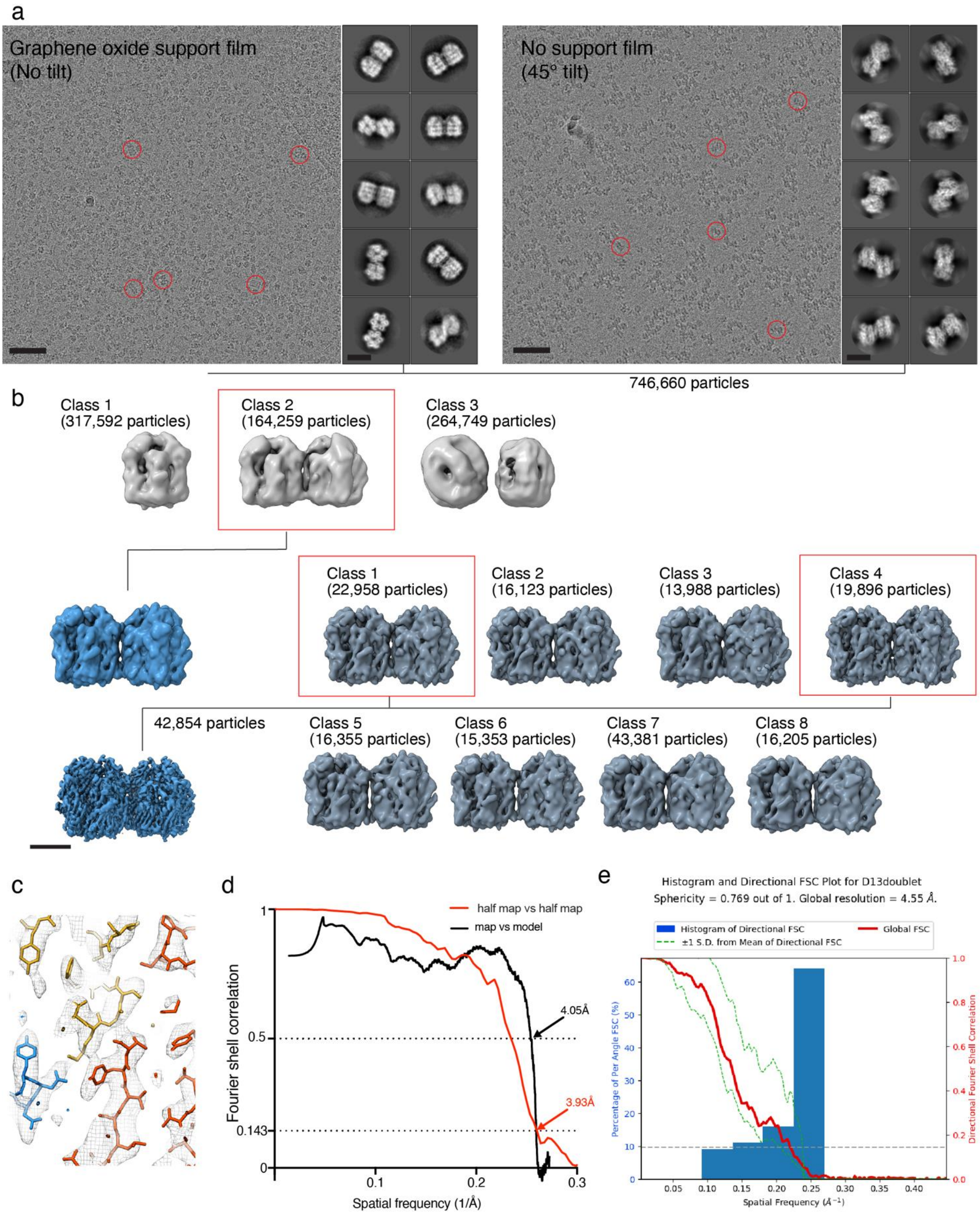


**Supplementary Figure 4. Cryo-EM image processing of D13 trimer doublet**

**a**, Representative cryo-electron micrographs and 2D class averages (right insets) generated from 227,275 and 519,385 images of the untiled (left) and tilted particles (right), respectively. A small subpopulation of D13 trimer doublets was found in the micrographs of the trimer sample on the graphene oxide film (representative particles indicated by red circle, left). Micrographs of the sample prepared without graphene oxide film were recorded while the microscope stage was tilted to 45° (representative particles indicated by red circle, right). The doublet particles imaged on

graphene oxide film had limited angular orientations, providing only top or side views. Additional data of tilted specimen imaged in the absence of graphene support film showed preferential particle orientation at the air-water interface. These images contained intermediate angular views that were critical for successful 3D reconstruction. **b**, Image processing workflow. A 3D classification without symmetry imposition was performed. Then, the particle images that belong to the best 3D class (red box) were used for 3D refinement, and further 3D classification without particle image alignment was performed. The final refined 3D reconstruction (contoured at 3.0σ) was generated from the images that belong to the 3D classes exhibiting clear structural details (red boxes). **c**, Example of fitted atomic model into the cryo-EM map. **d**, Fourier shell correlation between the half maps, and between the map and model-generated density, indicating approximately 3.9 Å resolution. **e**, 3D Fourier shell correlation plot indicating moderate anisotropic resolution of the reconstruction. Scale bars, 50 nm and 10 nm in the micrographs and 2D class averages in (a), respectively, and 5 nm in (b).

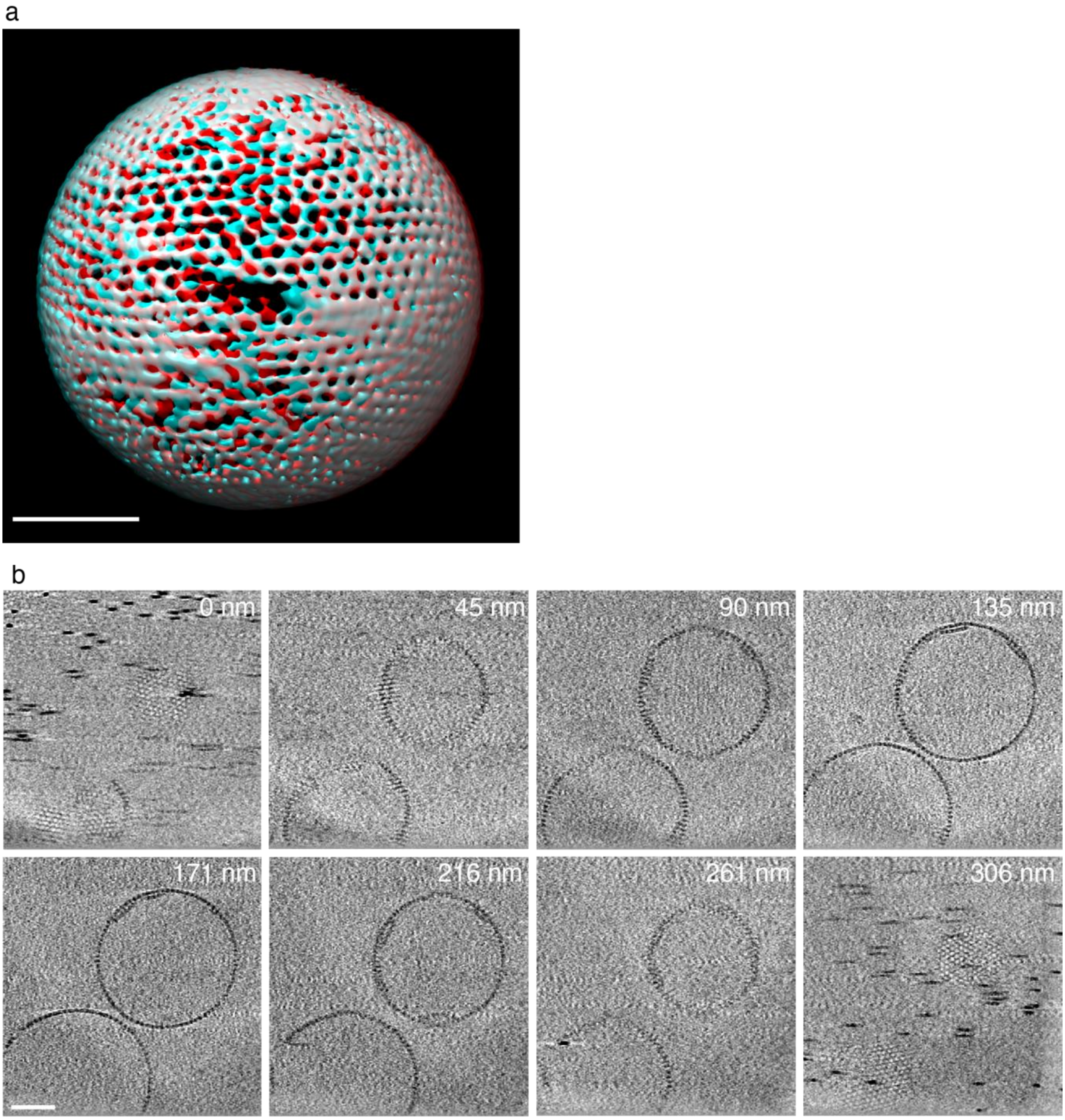


**Supplementary Figure 5. Cryo-electron tomography of spherical D13 self-assembly products**

**a,** A tomographic reconstruction of *in vitro* assembled spherical lattice formed by D13. The surrounding density was masked with a spherical mask for clear morphology representation. The anaglyph 3D image is colored for stereoscopic observation with red-cyan glasses. The cryo-ET reconstruction closely resembles deep-etch-EM of authentic VACV IV showing a continuous honeycomb-like D13 lattice with comparable size (Heuser, Journal of Cell Biology, vol.169(2) p269, 2005). **b**, Montage of slices (each representing an averaged projection of 10 sections corresponding to 23 Å thickness) through the reconstructed tomographic volume. The tomogram was created from a tilt series of 42 movie exposures collected at 3° intervals using 120 $e^-/Å^2$ total dose. The z-slice positions are indicated in top-right. Scale bars, 100 nm.

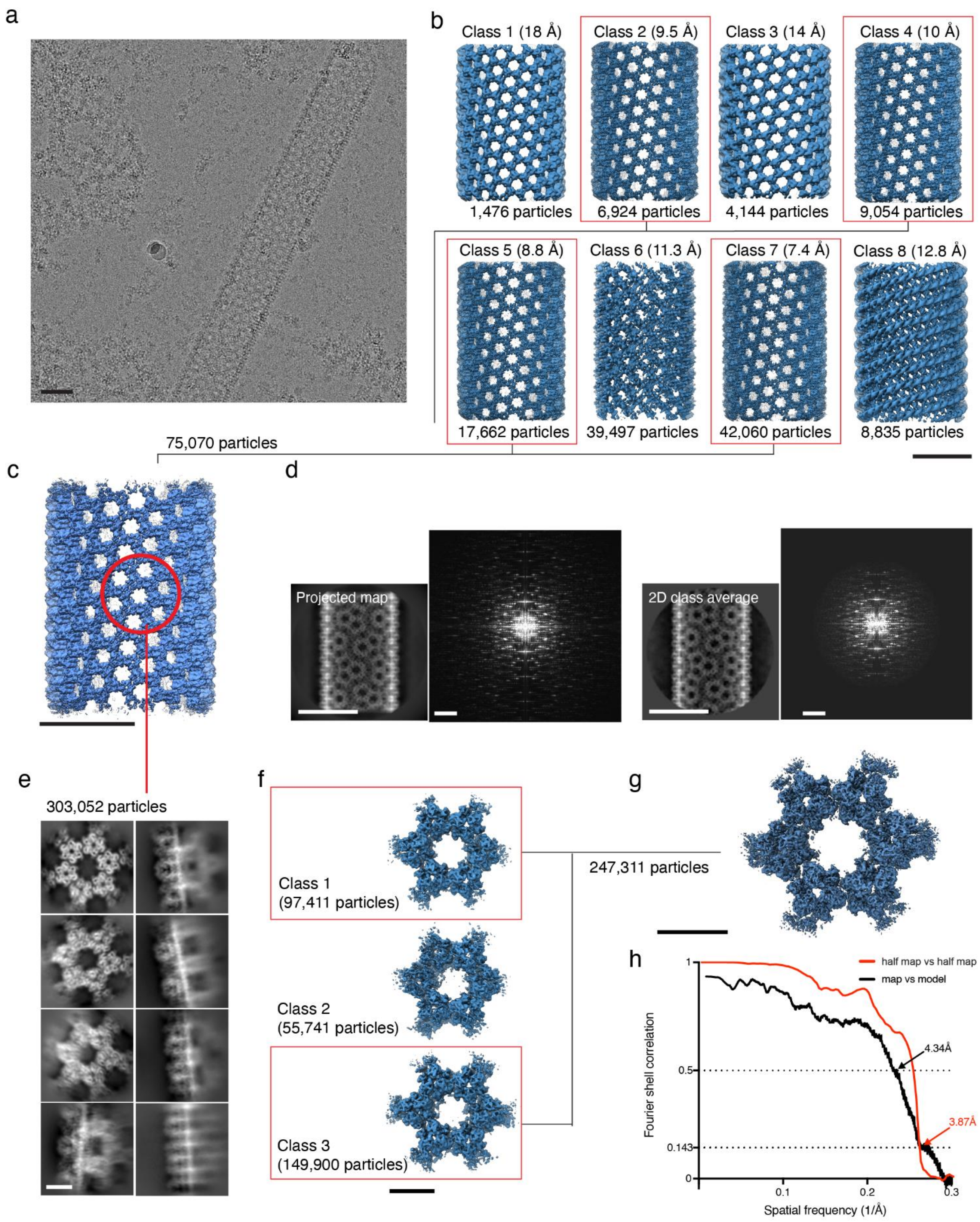


**Supplementary Figure 6. Cryo-EM image processing of the D13 tubular assembly**

**a**, Representative cryo-electron micrograph of the tubular D13 assembly in vitreous ice from total 7,621 micrographs. **b**, 3D classes of the assemblies displaying varying diameters. Diameters of the tubes were 92 nm, 89 nm, 96 nm, 85 nm, 90 nm, 84 nm, 88 nm and 92 nm, in the order from class 1 to class 8. **c**, Final 3D reconstruction at 7.3 Å resolution (isosurface contoured at 5.0σ), obtained from particle images that belong to good 3D classes (red boxes in b). **d**, Comparison between the projected 3D reconstruction and its power spectrum (left) and a class average and its power spectrum (right). **e**, Representative 2D class averages from the 303,052 signalsubtracted

sextet particle images. **f**, 3D classes of the sextet. The red circle in (**c**) marks the target region within the helical tube reconstruction used for (e) and (f). **g**, Final 3D reconstruction after signalsubtracted local alignment. **h**, the Fourier shell correlation plot between the half maps, and between the map and model-generated densities. Scale bars, 50 nm in (a-d), 5 $nm^{-1}$ in the power spectrum in (d), and 10 nm in (e-g), respectively.

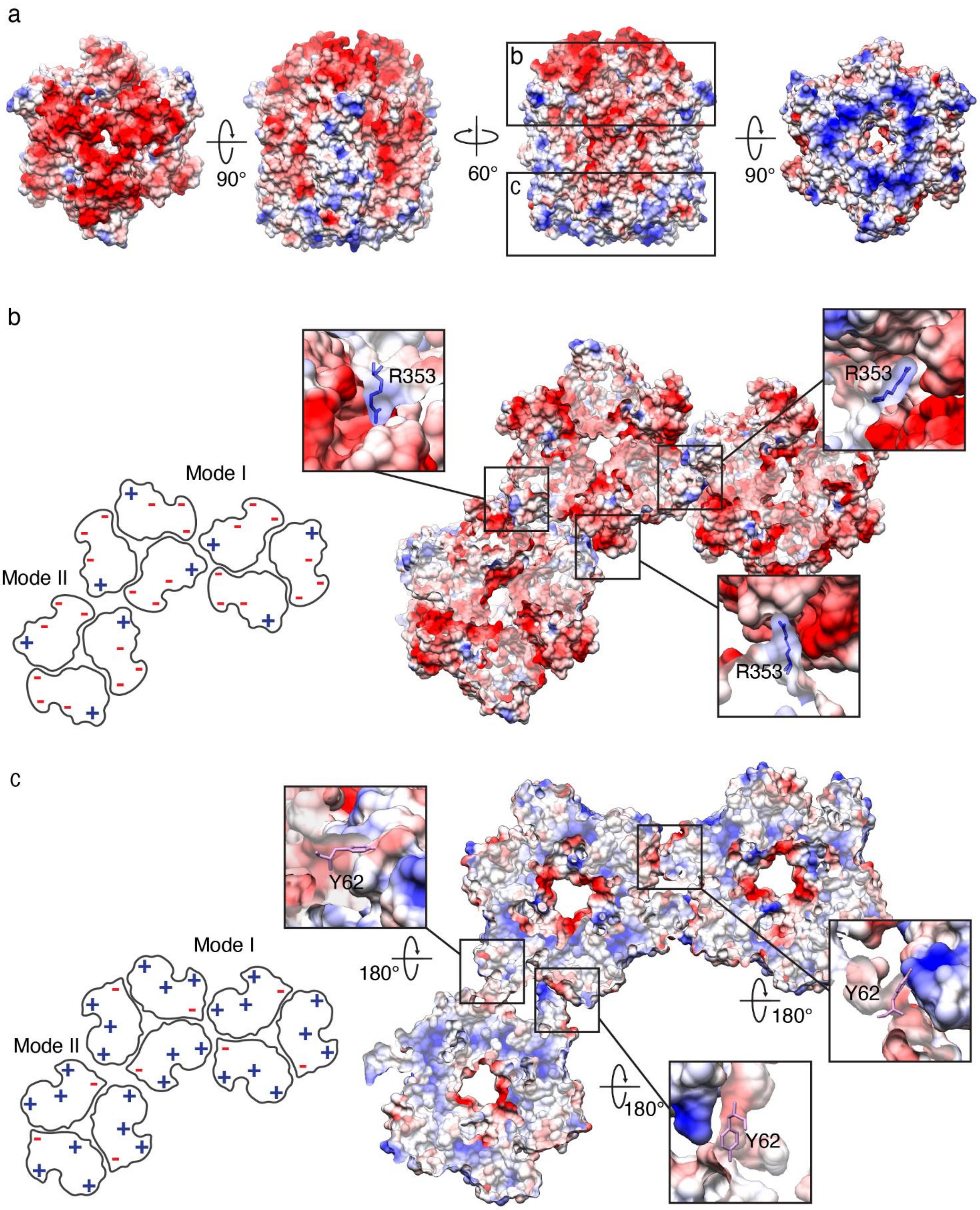


**Supplementary Figure 7. Intertrimer electrostatic interactions and critical residues in the D13 sextet**

**a**, Electrostatic potential map of the volume-rendered D13 trimer structure using coulombic surface coloring in UCSF Chimera. Overall, the membrane-distant region is predominantly negatively charged and the membrane-proximal part is positively charged. The charge distribution alternates along the circumference of the trimer, resulting in cogwheel-like intertrimer interfaces between oppositely charged surfaces. **b**, Alternating surface charge distribution at the head-to-head interface. Positively charged regions along the protrusion of the C-terminal jelly roll and the head

domain intermesh with the negatively charged grooves at the junction between the monomers. In both mode I and mode II arrangements, R353 extends toward its negatively charged partner residues at the interfaces. **c**, Alternating surface charge distribution at the base-to-base interface. Small patches of negatively charged regions that include Y62 are in contact with their positively charged counterparts.

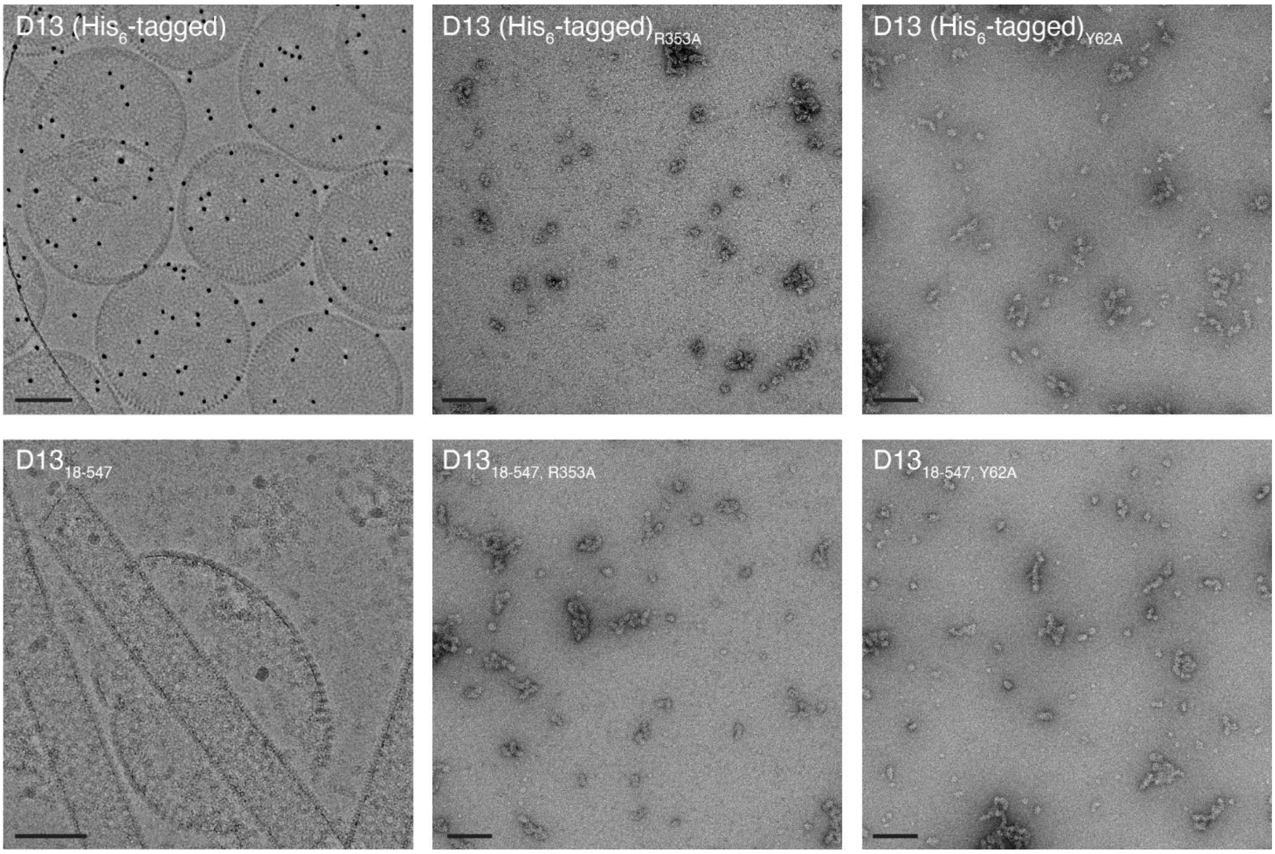


**Supplementary Figure 8. The effect of mutations on the D13 self-assembly**

Effect of mutations to critical residues involved in intertrimer electrostatic interactions on D13 self-assembly of the $His_6$-tagged wtD13 and the N-terminal deletion mutant $D13_{18\text{-}547}$. The residues R353 and Y62 were selected as targets for point mutations to alanine as they were deemed to be most critical in the stabilization of the intertrimer interactions in both mode I and mode II arrangements. Based on our cryo-EM structures, R353 either forms a salt bridge with D325 of the neighboring trimer in the mode I arrangement, or it is engaged in electrostatic interactions with nearby charged and polar residues in the mode II arrangement. Y62 forms cation-$\pi$ interactions with R498 and R446 in mode I and mode II arrangements, respectively. Without mutations, D13 assembles into spherical IV-like particles or tubes under low salt buffer condition (left panel, Supplementary Video 1 and Supplementary Fig. 5, 6). Under the same low salt condition, none of the mutants forms large assembly products. Instead, they form small random aggregates (middle and right panels). Assembly products and the mutation-induced random aggregates are shown by cryo-EM (left panel) and negative staining EM (middle and right panels), respectively. Representatives of a total of 40 and 7,621 cryo-electron micrographs of assembly products from $His_6$-tagged D13 and $D13_{18\text{-}547}$, respectively, are shown. Scale bars, 100 nm.

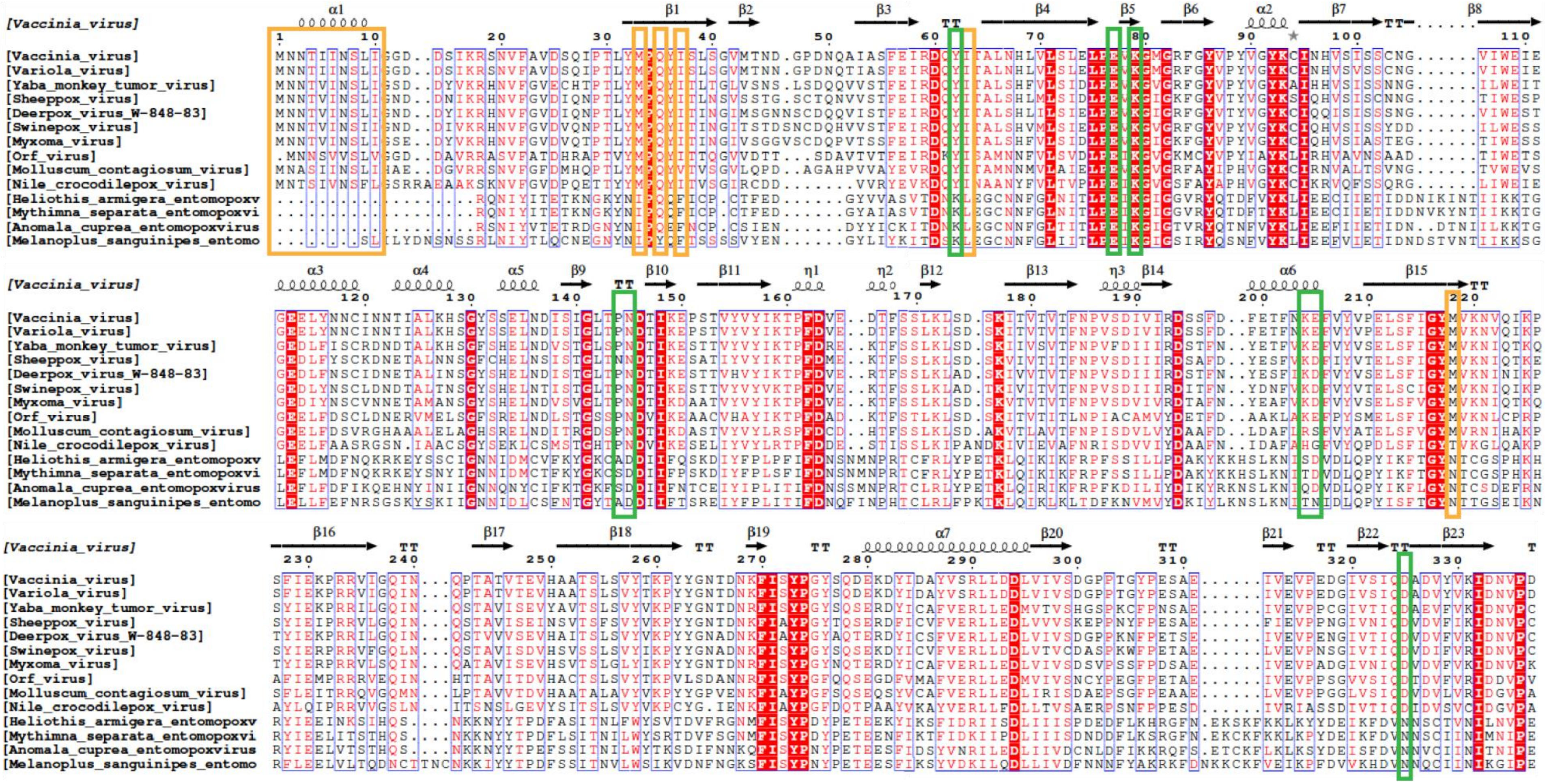
[Vaccinia_virus]
[Variola_virus]
[Yaba_monkey_tumor_virus]
[Sheeppox_virus]
[Deerpox_virus_W-848-83]
[Swinepox_virus]
[Myxoma_virus]
[Orf_virus]
[Molluscum_contagiosum_virus]
[Nile_crocodilepox_virus]
[Heliothis_armigera_entomopoxv
[Mythimna_separata_entomopoxvi
[Anomala_cuprea_entomopoxvirus
[Melanoplus_sanguinipes_entomo

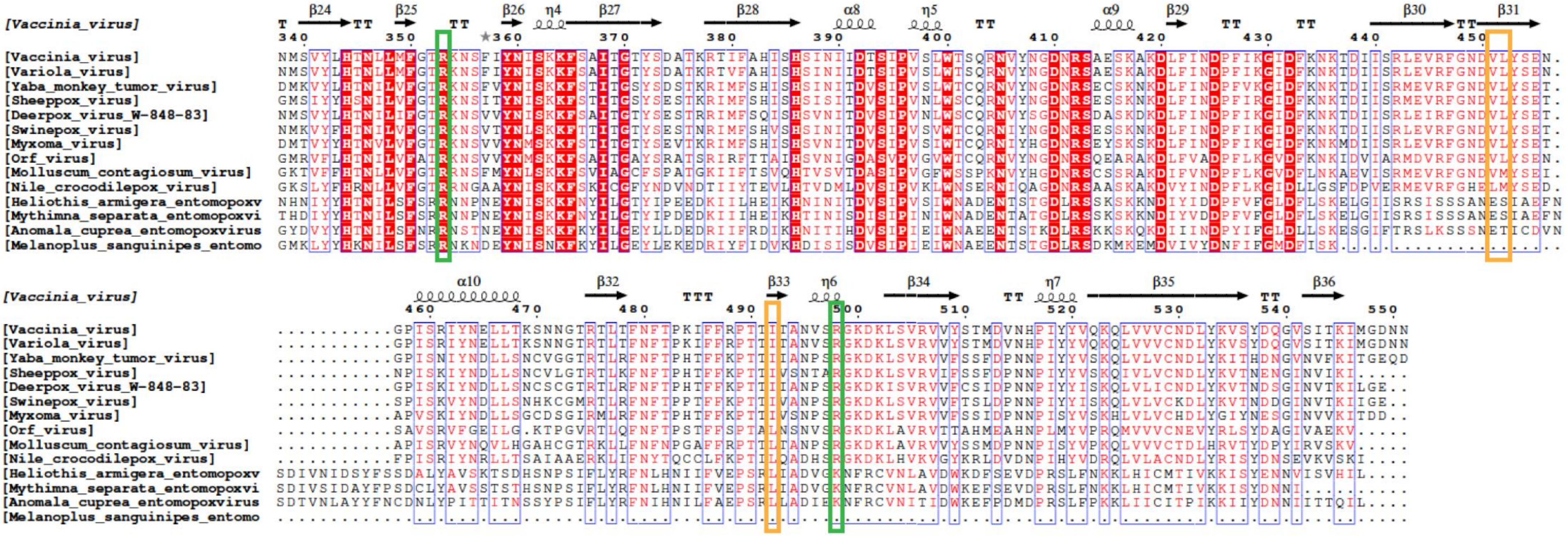

**Supplementary Figure 9. Sequence alignment between VACV D13 and the homologues from various poxvirus genera**

Sequences that correspond to the N-terminal helix and its binding pocket are framed in orange. Residues that are involved in intertrimeric interactions described in this study are framed in green. Entomopoxviruses (bottom 4 lines) do not contain a homolog sequence corresponding to the N-terminal helix.

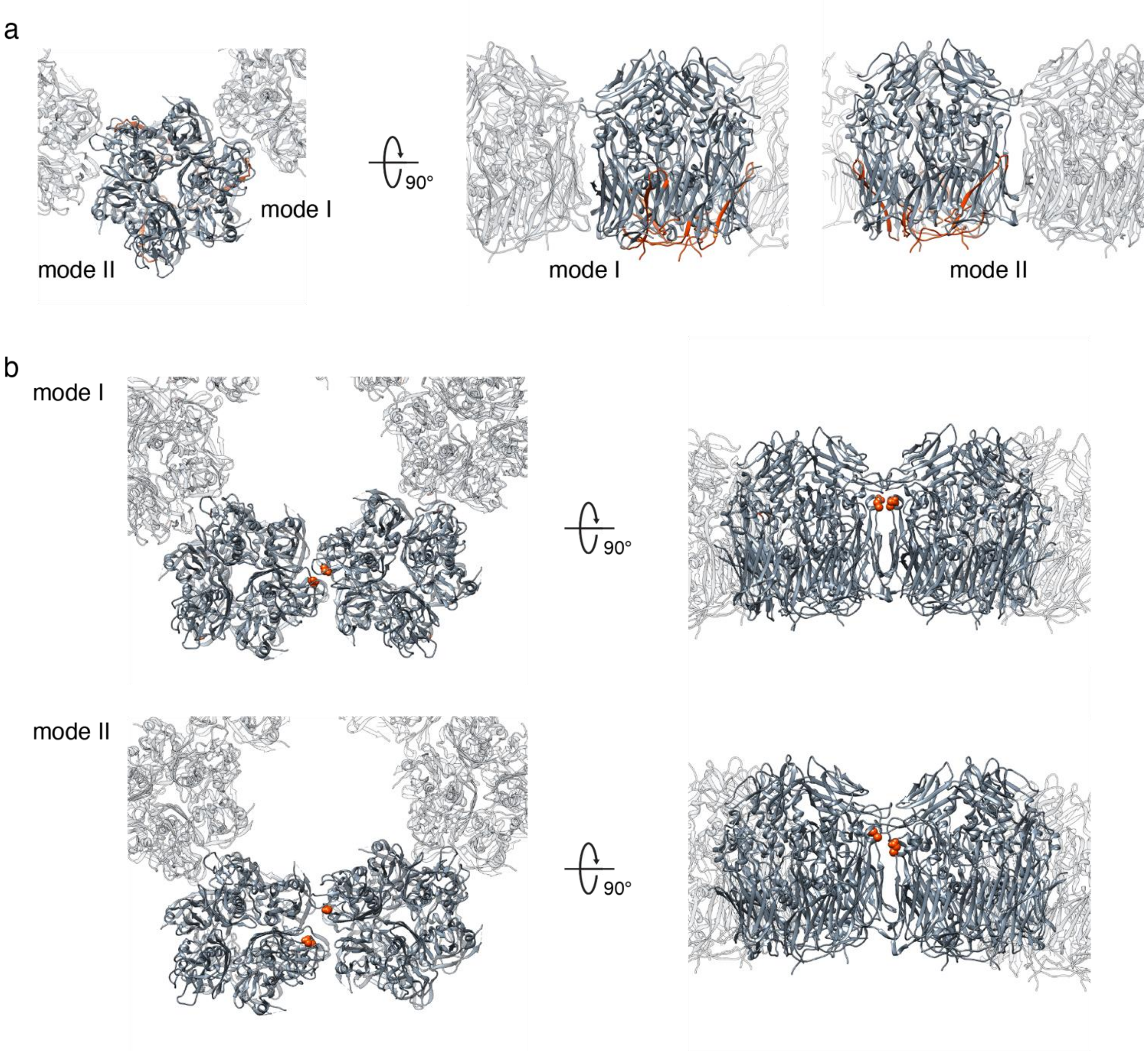


**Supplementary Figure 10. Intertrimer interactions and previously reported residues, mutations of which lead to rifampicin resistance or aberrant scaffold assembly**

**a**, Ribbon diagram of intertrimer interfaces of mode I and mode II arrangements. The rifampicin binding site and the mutation sites that confer rifampicin resistance are shown in red. In both intertrimer arrangements, these residues are distal from the intertrimer contacts. **b**, Mode I and II intertrimer arrangements with the D513 position highlighted as red spheres. Mutation of this residue to glycine leads to an aberrant phenotype of planar honeycomb-like assembly *in vivo* and to the abrogation of scaffold assembly. The residues are proximal in the mode I arrangement to the intertrimer interface, along the local 2-fold symmetry axis (upper panel). However, the interaction between these residues was not resolved clearly in our cryo-EM map. In the mode II arrangement, these residues are too far apart for electrostatic interaction (lower panel).

## Supplementary Table 1. Primer sequences

| Name | Sequences 5’- |
|---|---|
| $D13_{18\text{-}537}Fw$ | AATGGCGCCCGGTCTAATGTCTTCGCAGTCGATAGTC |
| $D13_{18\text{-}537}Rv$ | CCCAAGCTTTTAGTTATTATCTCCCATAATCTTGG |
| $D13_{R353A}Fw$ | GTTTGGAACAGCGAAAAATTCTTTTATATATAACATTTCTAAAAAG |
| $D13_{R353A}Rv$ | ATTAGCAGATTAGTATGAAGATAAAC |
| $D13_{Y62A}Fw$ | TAGGGATCAGGCGATTACTGCGC |
| $D13_{Y62A}Rv$ | ATTTCGAAGCTAGCGATAG |

**Supplementary Table 2. Cryo-EM data collection, refinement and validation statistics.**

| | wtD13 trimer (EMD-31949 / PDB 7VFD) | His$_6$-tagged D13 trimer (EMD-31950 / PDB 7VFE) | D13$_{18\text{-}547}$ trimer (EMD-31951 / PDB 7VFF) | D13 trimer doublet (EMD-31952 / PDB 7VFG) | D13 trimer tubular assembly (EMD-31953) | D13 trimer sextet (EMD-31954 / PDB 7VFH) |
|---|---|---|---|---|---|---|
| **Data collection and processing** | | | | | | |
| Magnification | 155,000 | 155,000 | 92,000 | 92,000 | 105,000 | 105,000 |
| Voltage (kV) | 300 | 300 | 200 | 200 | 300 | 300 |
| Electron exposure ($e^-$/ Å$^2$) | 50 | 50 | 50 | 50 | 50 | 50 |
| Defocus range (μm) | 0.5 – 1.5 | 0.5 – 1.5 | 0.6 – 1.2 | 0.5 – 5.0 | 0.5 – 2.5 | 0.5 – 2.5 |
| Pixel size (Å) | 0.518 | 0.518 | 1.12 | 1.12 | 1.39 | 1.39 |
| Symmetry imposed | C1, C3 | C3 | C3 | C2 | Helical (76.98 twist / 33.86 rise) | C1 |
| Initial particle images (no.) | 668,437 | 284,340 | 743,834 | 835,797 | 194,960 | 303,052 |
| Final particle images (no.) | 130,384 | 173,354 | 156,813 | 164,259 | 75,070 | 247,311 |
| Map resolution (Å) | 2.63 (C1), 2.25 (C3)* | 2.63 | 4.10 | 3.93 | 7.33 | 3.87 |
| FSC threshold | 0.143 | 0.143 | 0.143 | 0.143 | 0.143 | 0.143 |
| Map resolution range (Å) | 2.1 – 3.5 | 2.5 – 4.4 | 3.8 – 7.3 | 3.7 – 6.9 | 7.1 – 8.0 | 3.7 – 6.9 |
| **Model refinement** | | | | | | |
| Initial model used (PDB code) | 6BEI | 6BEI | 6BEI | 6BEI | | 6BEI |
| Model resolution (Å) | 2.27 | 2.64 | 4.06 | 4.05 | | 4.34 |
| | 0.5 | 0.5 | 0.5 | 0.5 | | 0.5 |
| Map sharpening B factor (Å$^2$) | -14.98 | -38.98 | -216.59 | -133.31 | | -8.42 |
| Model composition | | | | | | |
| Non-hydrogen atoms | 13245 | 12630 | 12579 | 25044 | | 75471 |
| Protein residues | 1623 | 1587 | 1581 | 3162 | | 9486 |
| B factors (Å$^2$) | 21.39 | 27.88 | 31.81 | 70.96 | | 27.25 |
| R.m.s. deviations | | | | | | |
| Bond length (Å) | 0.004 | 0.008 | 0.007 | 0.007 | | 0.003 |
| Bond angles (°) | 0.523 | 0.575 | 0.717 | 0.782 | | 0.610 |
| Validation | | | | | | |
| MolProbability score | 1.41 | 1.71 | 1.26 | 1.40 | | 1.27 |
| Clashscore | 3.69 | 2.86 | 2.36 | 3.80 | | 2.31 |
| Poor rotamers (%) | 0.60 | 3.08 | 0.41 | 0.45 | | 0.00 |
| Ramachandran plot | | | | | | |
| Favored (%) | 96.25 | 96.19 | 96.37 | 96.42 | | 96.19 |
| Allowed (%) | 3.75 | 3.81 | 3.63 | 3.58 | | 3.81 |
| Disallowed (%) | 0.00 | 0.00 | 0.00 | 0.00 | | 0.00 |

*Model refinement for wtD13 trimer was only performed using C3-symmetry-imposed map.
