# Supplementary material for: Assembly mechanism of the pleomorphic immature poxvirus scaffold
Source: Nat Commun. 2022 Mar 31;13:1704. doi: 10.1038/s41467-022-29305-5 (PMC8971458; doi:10.1038/s41467-022-29305-5)
Supplement: Supplementary file 3 — Description of Additional Supplementary Files [file 41467_2022_29305_MOESM3_ESM.pdf]

Description of Additional Supplementary Files

File name: Supplementary Movie 1

Description: **Electron tomography of immature poxvirus-like particles in amorphous ice**

Tomographic reconstruction of immature Vaccinia-virus like particles imaged in vitreous ice (cryo-ET). The sample was an assembly product of His_6-tagged D13 protein in sample buffer containing 50 mM Tris-HCl (pH 8.0) and 150 mM NaCl. The particles are characterized by spherical morphology with continuous curvature and a honeycomb-like pattern that resembles the authentic viral scaffold of immature Vaccinia virus. The video shows a planar section (x,y plane) moving through the volume along the z direction. Semiregular patches and aggregates near the level of the gold fiducial markers (black spheres) at z planes above or below the virus-like particles indicate that unassembled D13 can spontaneously assemble at the airwater interface of the liquid sample before freezing. Parts of doublelayered shells are also visible in the reconstruction. The opposite charges at the head and base of the D13 trimers (Supplementary Data Fig. 6a) may drive the stacking of these shells in the absence of an underlying lipid membrane.
