# Supplementary material for: Assembly mechanism of the pleomorphic immature poxvirus scaffold
Source: Nat Commun. 2022 Mar 31;13:1704. doi: 10.1038/s41467-022-29305-5 (PMC8971458; doi:10.1038/s41467-022-29305-5)
Supplement: Supplementary file 5 — Reporting Summary [file 41467_2022_29305_MOESM5_ESM.pdf]

Corresponding author(s): Matthias Wolf, Jaekyung Hyun

Last updated by author(s): 2022 02 15

Reporting Summary

Nature Portfolio wishes to improve the reproducibility of the work that we publish. This form provides structure for consistency and transparency in reporting. For further information on Nature Portfolio policies, see our Editorial Policies and the Editorial Policy Checklist.

Statistics

For all statistical analyses, confirm that the following items are present in the figure legend, table legend, main text, or Methods section.

n/a	Confirmed	
☒	☐	The exact sample size (*n*) for each experimental group/condition, given as a discrete number and unit of measurement
☒	☐	A statement on whether measurements were taken from distinct samples or whether the same sample was measured repeatedly
☒	☐	The statistical test(s) used AND whether they are one- or two-sided<br>*Only common tests should be described solely by name; describe more complex techniques in the Methods section.*
☒	☐	A description of all covariates tested
☒	☐	A description of any assumptions or corrections, such as tests of normality and adjustment for multiple comparisons
☒	☐	A full description of the statistical parameters including central tendency (e.g. means) or other basic estimates (e.g. regression coefficient) AND variation (e.g. standard deviation) or associated estimates of uncertainty (e.g. confidence intervals)
☒	☐	For null hypothesis testing, the test statistic (e.g. *F*, *t*, *r*) with confidence intervals, effect sizes, degrees of freedom and *P* value noted<br>*Give P values as exact values whenever suitable.*
☒	☐	For Bayesian analysis, information on the choice of priors and Markov chain Monte Carlo settings
☒	☐	For hierarchical and complex designs, identification of the appropriate level for tests and full reporting of outcomes
☒	☐	Estimates of effect sizes (e.g. Cohen's *d*, Pearson's *r*), indicating how they were calculated

Our web collection on statistics for biologists contains articles on many of the points above.

Software and code

Policy information about availability of computer code

Data collection	Thermo Fisher Scientific EPU 2.8 (Titan Krios) and EPU 2.12 (Talos Arctica) were used for single particle cryo-EM data collection.<br>Thermo Fisher Scientific Tomography4 was used for cryo-electron tomography data collection.
Data analysis	Relion 3.1.0, cisTEM 1.0.0, motioncor2 v1.4.2, ctffind 4.1.9, IMOD 4.11 were used for cryo-EM data processing.<br>Phenix 1.18, Coot 0.9.6 were used for structure modeling.<br>UCSF Chimera 1.13.1, UCSF Chimera X 0.93, PyMol 2.4.0 were used for structure visualization and figure preparation.

For manuscripts utilizing custom algorithms or software that are central to the research but not yet described in published literature, software must be made available to editors and reviewers. We strongly encourage code deposition in a community repository (e.g. GitHub). See the Nature Portfolio guidelines for submitting code & software for further information.

Data

Policy information about availability of data

All manuscripts must include a data availability statement. This statement should provide the following information, where applicable:

- Accession codes, unique identifiers, or web links for publicly available datasets
- A description of any restrictions on data availability
- For clinical datasets or third party data, please ensure that the statement adheres to our policy

The atomic coordinates of the cryo-EM based model have been deposited into the RCSB Protein Data Bank (PDB ID: 7VFD, 7VFE, 7VFF, 7VFG, 7VFH).
The cryo-EM maps have been deposited into the EM Data Bank (access codes: EMD-31949, EMD-31950, EMD-31951, EMD-31952, EMD-31953, EMD-31954).

Field-specific reporting

Please select the one below that is the best fit for your research. If you are not sure, read the appropriate sections before making your selection.

☒ Life sciences ☐ Behavioural & social sciences ☐ Ecological, evolutionary & environmental sciences

For a reference copy of the document with all sections, see nature.com/documents/nr-reporting-summary-flat.pdf

Life sciences study design

All studies must disclose on these points even when the disclosure is negative.

Sample size	Cryo-EM data processing statistics are provided in Supplementary Table 2.
Data exclusions	All acquired digital micrographs were analyzed initially. Particle locations were automatically identified from each micrograph using a template produced from manually picked particle images. This procedure always results in a certain amount of false positives (e.g. images of non-protein and high contrast background objects, or contamination). A series of classifications, both 2D and 3D, were used to exclude these false positive particle images from the subsequent processing workflow. This procedure is common practice for cryo-EM image processing and is described in the Cryo-EM image processing Methods section. Cryo-EM Statistics are provided in Supplementary Table 2.
Replication	For every representative electron micrographs shown in the figure, the number of total micrographs in the dataset has been provided in the relevant figure legends.
Randomization	For independent single-particle alignment in cryo-EM 3D refinement, datasets were split into two subsets which were processed independently (so-called "gold standard").
Blinding	Unlike in crystallography (e.g. cross-validated R-factor), there are no well-established procedures for blinding in cryo-EM. We used reference-free 2D classification and ab-initio 3D reconstruction in Relion / cisTEM without any external reference.

Reporting for specific materials, systems and methods

We require information from authors about some types of materials, experimental systems and methods used in many studies. Here, indicate whether each material, system or method listed is relevant to your study. If you are not sure if a list item applies to your research, read the appropriate section before selecting a response.

Materials & experimental systems

n/a	Involved in the study	
☒	☐	Antibodies
☒	☐	Eukaryotic cell lines
☒	☐	Palaeontology and archaeology
☒	☐	Animals and other organisms
☒	☐	Human research participants
☒	☐	Clinical data
☒	☐	Dual use research of concern

Methods

n/a	Involved in the study	
☒	☐	ChIP-seq
☒	☐	Flow cytometry
☒	☐	MRI-based neuroimaging
